# The Molecular Mechanism of Ion Selectivity in Nanopores

**DOI:** 10.3390/molecules29040853

**Published:** 2024-02-14

**Authors:** Yan-Nan Chen, Yu-Zhen Liu, Qiang Sun

**Affiliations:** Key Laboratory of Orogenic Belts and Crustal Evolution, The School of Earth and Space Sciences, Ministry of Education, Peking University, Beijing 100871, China; 2301210129@stu.pku.edu.cn (Y.-N.C.); liuyuzhen@stu.pku.edu.cn (Y.-Z.L.)

**Keywords:** ion selectivity, nanopore, water, hydrogen bondings, hydrophobic interactions

## Abstract

Ion channels exhibit strong selectivity for specific ions over others under electrochemical potentials, such as KcsA for K^+^ over Na^+^. Based on the thermodynamic analysis, this study is focused on exploring the mechanism of ion selectivity in nanopores. It is well known that ions must lose part of their hydration layer to enter the channel. Therefore, the ion selectivity of a channel is due to the rearrangement of water molecules when entering the nanopore, which may be related to the hydrophobic interactions between ions and channels. In our recent works on hydrophobic interactions, with reference to the critical radius of solute (Rc), it was divided into initial and hydrophobic solvation processes. Additionally, the different dissolved behaviors of solutes in water are expected in various processes, such as dispersed and accumulated distributions in water. Correspondingly, as the ion approaches the nanopore, there seems to exist the “repulsive” or “attractive” forces between them. In the initial process (<Rc), the energy barrier related to “repulsive” force may be expected as ions enter the channel. Regarding the ion selectivity of nanopores, this may be due to the energy barrier between the ion and channel, which is closely related to the ion size and pore radius. Additionally, these may be demonstrated by the calculated potential mean forces (PMFs) using molecular dynamics (MD) simulations.

## 1. Introduction

Ion channels with nanoscale pores are specialized membrane proteins. They play a vital role in allowing specific ion movements across the cell membrane, which is known as ion selectivity and is a fundamental property defining ion channel function. From this, they perform key functions in cell behavior, such as electrical excitability, regulation of cell volume, regulation of cytoplasmic ion concentration and pH, and hormone and neurotransmitter secretion [1]. Additionally, due to analogies with biological membranes, much attention has been paid to the selectively permeable membranes with sub-nm pores because of their potential applications, such as in water filtration, molecular separation, and the desalination of seawater [2,3,4]. Therefore, it is important to investigate the mechanism of ion selectivity in nanopores.

In fact, the current knowledge on such a selective process was laid down by MacKinnon’s [5,6,7] group in the reports on the structure of KcsA, a potassium channel from Streptomyces lividans. Potassium (K^+^) channels are tetrameric membrane-spanning proteins that provide a selective pore for the conductance of K^+^ across the cell membranes. This structure revealed that, in the narrow pore region forming the selectivity filter, the backbone carbonyl oxygen atoms (as well as threonine hydroxyls) of the TVGYG sequence form a series of cage-like K^+^ binding sites, termed S1–S4, and a non-cage-forming S0 site. Under physiological conditions, the channels are K^+^-selective protein pores in cell membranes, which display up to a 1000-fold preference for K^+^ over Na^+^ [8,9], despite the fact that the two ions are both spherical in nature, have the same charge, and differ in atomic radii by only 0.38 Å. Therefore, the selectivity filter is the functional unit that allows K^+^ channels to distinguish potassium (K^+^) and sodium (Na^+^) ions.

Many experimental and theoretical works have been conducted on the ion selectivity of the KcsA channel [10,11,12,13,14,15,16,17,18,19,20,21,22,23,24,25,26,27,28,29,30,31]. Additionally, the selectively permeable graphene nanopores have also been attracting much attention to unravel the mechanism of ion selectivity in filters [32,33]. These show that, under transmembrane voltage bias, a nanopore containing four carbonyl groups to mimic the selectivity filter of the KcsA K^+^ channel preferentially conducts K^+^ over Na^+^ [32]. From the free energy calculations, these indicate that, compared to K^+^, the smaller radius of Na^+^ leads to a significantly higher free energy barrier in the nanopore of a certain size [32]. Additionally, it indicates that the ion selectivity of such biomimetic graphene nanopores can be simply controlled by the size of the nanopore [33], and the graphene nanopores with a distance of about 3.9 Å between two neighboring oxygen atoms present excellent ion selectivity for Na^+^ and K^+^ ions.

So far, various models have been proposed to understand the mechanism of ion selectivity of channels. The classical “snug-fit” mechanism [34] suggested that the permeating ions would need to fit snugly in a rigid selectivity filter, which would allow the coordinating residues to solvate the preferred ions and provide a parsimonious explanation for the exclusion of smaller ions, such as Na^+^ in potassium channels. In the field strength model of Eisenman [35,36], it is suggested that ion selectivity may be regulated by the strength of the interactions between the ions and the residues. From Noskov et al.’s study [37], as residues are packed as tightly as they are in the selectivity filter, it is necessary to consider not only the ion–residue interactions but also the residue–residue interactions. Therefore, they proposed a “carbonyl-repulsion” mechanism whereby the increased repulsion upon coordinating the smaller Na^+^ leads to K^+^/Na^+^ selectivity in K^+^ channels. In addition, the “over-coordination hypothesis” is proposed by several groups [38,39,40,41]. This means that each cation is coordinated by more residues than it would experience in bulk water. Therefore, ion selectivity is due to altering the number of residues, not their field strength. From the above, further study is necessary to understand the mechanism of ion selectivity in nanopores.

In fact, as K^+^ passes through the KcsA filter, the ion must be almost completely dehydrated within the structural confinement of a narrow pore, which undoubtedly leads to the changes in hydrogen bondings of water. The ion selectivity of nanopores may be related to the rearrangement of water molecules when the ion enters the nanopore. In combination with our recent studies [42,43,44,45,46,47,48,49,50,51], this is closely related to hydrophobic interactions, which may be reasonably ascribed to the structural competition between interfacial and bulk water.

Based on thermodynamic analysis and MD simulations, this work is devoted to investigating the molecular mechanism of ion selectivity in nanopores. In our recent studies [47,48,49,50,51], hydration free energy was derived and applied to study the origin of hydrophobic effects. Hydration free energy is dependent on the solute size. With reference to the critical radius of solute (Rc), this is divided into initial and hydrophobic solvation processes, corresponding to the different dissolving behaviors of solutes in water, such as dispersed and accumulated distributions in solution [47,48,49,50,51]. Correspondingly, as the ion approaches the nanopore, “repulsive” or “attractive” forces may be expected between them. In the initial process (<Rc), the energy barrier is expected as ions enter the channel. The ion selectivity in nanopores may be related to the energy barrier when the ion enters the channel. It is found that the energy barrier may be dependent on the ion size and pore radius. Additionally, the barrier may also be modulated by the direct interactions between the ions and nanopores.

## 2. Results and Discussion

### 2.1. Thermodynamics of Ion Selectivity

In thermodynamics, Gibbs free energy (Δ*G*) can be utilized to investigate whether a process is likely to occur. As the solutes are embedded into water, this includes the solute–solute, the solute–water, and the water–water interactions. The total Gibbs energy may be expressed as

(1) $$\Delta G_{Total}=\Delta G_{Solute-solute}+\Delta G_{Solute-water}+\Delta G_{Water-water}$$

In fact, this may be applied to investigate the ion selectivity of channels as the ions pass through the filters.

For the selectivity filter of the KcsA channel, the ion selectivity represents a competition of K^+^ and Na^+^ for a binding site in the filter. The selectivity for K^+^ over Na^+^ is due to the difference in free energies of the ions in the pore, which departs from the corresponding difference in the bulk solution. It follows that the ion selectivity of binding is fundamentally governed by differences in relative free energies [31], and the ion selectivity can be stated from the point of view of thermodynamic equilibrium as

(2) $$\Delta \Delta G\left[K^{+}\to Na^{+}\right]=\Delta G_{site}\left[K^{+}\to Na^{+}\right]-\Delta G_{bulk}\left[K^{+}\to Na^{+}\right]$$

Thermodynamically, it can be understood to allow the preferential permeation of K^+^ over Na^+^ by providing an unfavorable environment for Na^+^ in the presence of K^+^.

Regarding the KcsA K^+^ channel, there are mainly O-atom quaternion rings with three sizes: 3.12/3.33 Å, 3.89 Å, and 4.22 Å. When the ions permeate the nanopore channels, one unambiguous observation based on the crystallographic X-ray structures of K^+^ channels is that the K^+^ ions must become essentially dehydrated to pass through the narrow selectivity filter [6] and directly interact with the filter. Therefore, as the ions pass through the nanopore channels, this undoubtedly leads to the changes in water structure.

As the ion permeates the nanopore, the ion selectivity of the nanopore may be closely related to not only the changes in hydrogen bondings in water, but also the direct interactions between the ion and channel. Therefore, the ion selectivity of the nanopore may be related to various interactions, such as those due to the solute–solute, solute–water, and water–water interactions. In fact, before the ions are affected by the interactions between ions and the channel, it is necessary for the ions to approach the filter. This may be due to the rearrangement of water molecules as ions approach the nanopore, which is related to ΔG_Water-water_ and ΔG_Solute-water_. Therefore, it is important to investigate the structure of liquid water, and the effects of dissolved solute on the water structure.

Numerous experimental and theoretical works have been conducted to investigate the structure of liquid water. To date, a variety of structural models for water have been proposed, broadly categorized into two groups: (a) mixture models and (b) distorted hydrogen bond or continuum models [52]. The mixture model postulates the concurrent existence of two distinct types of structures. In the latter model, this implies that water consists of a random, three-dimensional network of hydrogen bonds with a wide distribution of O-H∙∙∙O hydrogen bond angles and distances. However, unlike in the mixture models, the water networks cannot be “broken” or separated into distinct molecular species.

The OH vibrations are sensitive to hydrogen bondings and are employed to study the structure of water. Based on the dependence of OH vibrations on water clusters ((H_2_O)_n_) [42,43,44], it is found that, as three-dimensional hydrogen bondings emerge (*n* ≥ 6), OH vibrations are predominantly influenced by the local hydrogen bondings (within the first shell) of water molecules, and the effects of hydrogen bonding beyond the first shell on OH vibrations are weak. Hence, different OH vibrations may reasonably be attributed to the OH vibrations engaged into various local hydrogen-bonded networks of a water molecule.

Under ambient conditions, the Raman OH stretching bands of water can be fitted into five sub-bands. These may be attributed to OH vibrations involved in various local hydrogen bonds, including DDAA (double donor-double acceptor, tetrahedral hydrogen bonding), DDA (double donor-single acceptor), DAA (single donor-double acceptor), and DA (single donor-single acceptor) hydrogen bondings, respectively. In ambient water, a water molecule interacts with neighboring water molecules (the first shell) through various local hydrogen-bonded networks [44]. It is different from the mixture and continuum structural models of water. Additionally, under ambient conditions, the local hydrogen-bonded networks of a water molecule may be influenced by factors such as temperature, pressure, dissolved salt, and confined environments.

When a solute is embedded in water, an interface appears between the solute and water, which undoubtedly affects the structure of liquid water. As the OH vibration is primarily influenced by the local hydrogen bondings of a water molecule, the dissolved solute mainly affects the structure of interfacial water (the topmost water layer at the solute/water interface). In theory, vibrational sum frequency generation (SFG) spectroscopy is a technique selectively applicable to interfaces and is widely used to examine the structure of the air/water interface. Based on the SFG study [45] on the air/water interface, it is observed that, in contrast to bulk water, no DDAA (tetrahedral) hydrogen bondings are present in interfacial water.

The solute mainly affects the hydrogen bondings of interfacial water. The formation of the solute/water interface is related to the loss of DDAA hydrogen bonding in interfacial water layer. Once the ratio of the interfacial water layer to the volume is established, it can be employed to calculate the Gibbs free energy between solute and water (interfacial water). Therefore, ΔG_Solute-water_ can reasonably be expressed as

(3) $$\Delta G_{Solute-water}=n_{HB}\cdot \Delta G_{DDAA}\cdot R_{Interfacial\;\;water}$$

where R_Interfacial water_ represents the ratio of interfacial to bulk water, ∆G_DDAA_ is the Gibbs energy of DDAA hydrogen bondings, and n_HB_ means the average hydrogen bondings of a water molecule. For DDAA hydrogen bonidngs, n_HB_ is equal to 2.

The hydration free energy refers to the change in Gibbs energy when an ion (or molecule) is transferred from a vacuum (or the gas phase) to a solvent. Thermodynamically, the process of inserting a hard sphere into water is equivalent to the formation of an empty spherical cavity in water. After the solute is regarded as an ideal sphere, the hydration free energy may be expressed as [47]

(4) $$\begin{array}{l} \Delta G_{Hydration}=\Delta G_{Water-water}+\Delta G_{Solute-water}\\ \;\;\;\;=\Delta G_{Water-water}+\frac{8\;\cdot \;\Delta G_{DDAA}\;\cdot \;r_{H2O}}{R} \end{array}$$

where r_H2O_ is the average radius of an H_2_O molecule, and R represents the radius of the solute. At ambient conditions, the Gibbs energy of DDAA hydrogen bondings is determined to be −2.66 kJ/mol [47].

**Figure 1:**
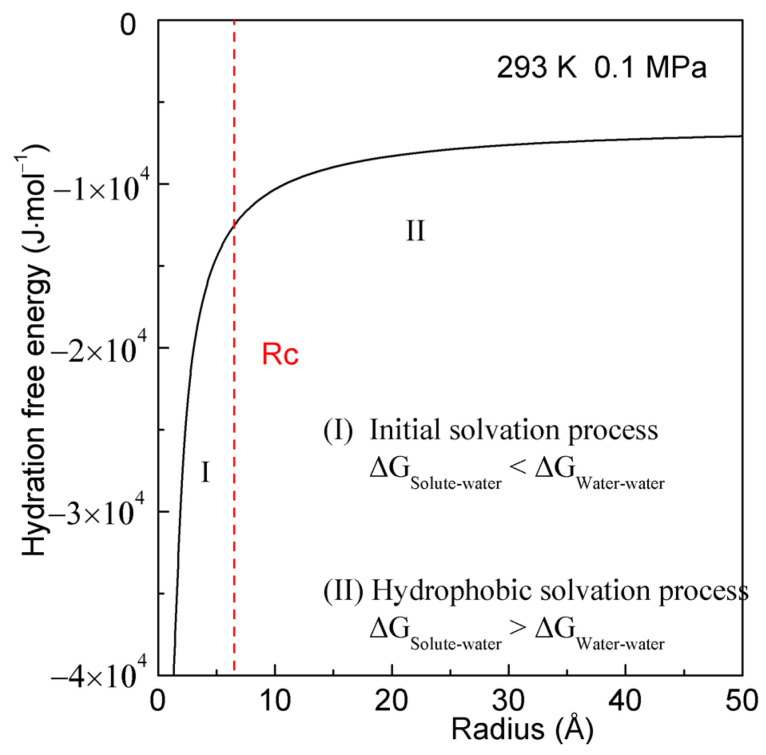
Hydration free energy at 293 K and 0.1 MPa. With reference to R_c_, it can be divided into initial and hydrophobic solvation processes. The R_c_ is drawn as a dashed line. (I) Initial solvation process. (II) Hydrophobic solvation process.

In principle, the lower the hydration free energy, the more thermodynamically stable the system becomes. The hydration free energy is the sum of Gibbs energy of pure water (ΔG_Water-water_) and that of interfacial water (ΔG_Solute-water_) related to the dissolved solute. Therefore, the structural transition is expected when ΔG_Water-water_ equals ΔG_Solute-water_ (Figure 1).

(5) $$\Delta G_{Water-water}=\Delta G_{Solute-water}\;\;\;\;\;\;\left(R_{c}=\frac{8\cdot \Delta G_{DDAA}\cdot r_{H2O}}{\Delta G_{Water-water}}\right)$$

where R_c_ represents the critical radius of solute. At 293 K and 0.1 MPa, R_c_ is calculated to be 6.5 Å. With increasing the solute size (or concentrations), it is divided into the initial and hydrophobic solvation processes [47,48]. Additionally, the Gibbs free energy of interfacial water (ΔG_Solute-water_) is inversely proportional to the radius of solute (or the ratio of surface area to volume of solute). As the solute size (or solute concentrations) increases, different dissolved behaviors can be expected for various processes (Figure 1) [47,48].

In the initial solvation process (∆G_Solute-water_ < ∆G_Water-water_, both of which are negative), the hydration free energy is dominated by the Gibbs energy of interfacial water (ΔG_Solute-water_). To achieve greater thermodynamic stability, the solutes tend to be dispersed in water to maximize ΔG_Solute-water_. Consequently, water molecules are expected to exist between solutes. In other words, it appears that there are “repulsive” forces between the solutes as they are brought closer together. In the hydrophobic solvation process (∆G_Solute-water_ > ∆G_Water-water_), the hydration free energy is primarily governed by the Gibbs energy of water (ΔG_Water-water_). To maximize the Gibbs energy of water, the dissolved solutes are expected to be aggregated to minimize the interfacial water. It seems that there are “attractive” forces between the dissolved solutes.

Hydrophobic effects are generally defined as the tendency of non-polar molecules (or molecular surfaces) to aggregate in an aqueous solution. In our recent investigations [47,48] on hydrophobic interactions, they were reasonably characterized as the tendency to minimize the ratio of the surface area to the volume of the solutes in order to maximize the hydrogen bondings of water. Of course, this is due to the dissolved solute mainly affecting the structure of interfacial water, and the hydrogen bondings of interfacial water are weaker than bulk water. Additionally, the origin of hydrophobic interactions arises from the structural competition between interfacial and bulk water [47,48].

The Gibbs free energy between solute and water (ΔG_Solute-water_) is closely related to the water molecules of the interfacial water layer. When two solutes are embedded into water, the interfacial water of two solutes may be inversely proportional to the distance between them. Therefore, ΔG_Solute-water_ is reasonably expressed as

(6) $$\Delta G_{Solute-water}\propto 1/R_{Solute-solute}$$

where R_Solute-solute_ means the distance between solutes. As two identical spheres are dissolved in ambient water, the R_c_ is 3.25 Å [48]. As the distance between solutes is larger or less than R_c_, hydrophobic interactions between solutes may be regarded as the “attractive” or the “repulsive” forces [48].

**Figure 2:**
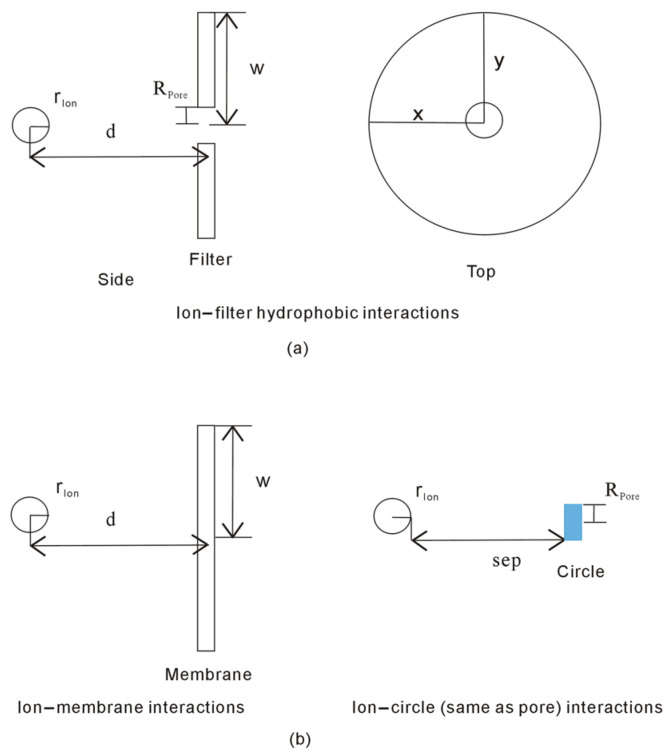
(**a**) To investigate the ion selectivity of nanopore, the ion moves perpendicularly to the filter embedded with a nanopore with radius R. Both side and top views are shown. (**b**) In thermodynamics, the Gibbs free energy (ΔG_Ion-filter_) related to the ion and filter equals the difference in Gibbs energy between the ion and membrane (ΔG_Ion-membrane_), and that between the ion and filled circle with radius R (ΔG_Ion-circle_). Additionally, d is the distance between the centers of ion and filter, and sep means the separation between the ion and nanopore.

To investigate the ion selectivity of nanopores, the test solute is a sphere, and the target filter is the membrane embedded with a nanopore (Figure 2). As the distance between the ion and filter is decreased, the hydration free energy may reasonably be expressed as

(7) $$\Delta G_{Hydration}=\Delta G_{Water-water}+\iint \Delta G_{Ion-filter}dxdy$$

where ΔG_Ion-filter_ is the Gibbs energy of interfacial water arising from the ion and filter, which is inversely proportional to the distance between them, ∝1/d_Ion-filter_, where d_Ion-filter_ is the distance between the ion and filter (Figure 2).

In fact, as the nanopore is filled with a circle with corresponding radius R, the filter becomes the membrane with the same size as the filter (Figure 2). Therefore, as the ion approaches the filter, the Gibbs free energy (ΔG_Ion-filter_) related to the ion and filter equals the difference in Gibbs energy between the ion and membrane (ΔG_Ion-membrane_), and that between the ion and filled circle with radius R (ΔG_Ion-circle_) (Figure 2), which may be expressed as

(8) $$\Delta G_{Ion-filter}=\Delta G_{Ion-membrane}-\Delta G_{Ion-circle}$$

As the ion enters the nanopore, the interfacial water molecules related to the ion and nanopore are changed into bulk water, which may be related to the ΔG_Ion-circle_.

In comparison with the membrane, the test ion is treated as a point after the size of the ion is ignored. Additionally, the ion is restrained to perpendicularly move to the center of the membrane. Therefore, the Gibbs energy between the ion and membrane (ΔG_Ion-membrane_) may mathematically be determined as (Figure 2)

(9) $$\Delta G_{Ion-membrane}\propto 1/d_{Ion-membrane}=\pi \cdot \left[\sqrt{w^{2}+d^{2}}-d\right]$$

where w is the radius of membrane, and d means the distance between the centers of the ion and membrane. In addition, ΔG_Ion-membrane_ is higher than ΔG_Water-water_ (ΔG_Ion-membrane_ >ΔG_Water-water_), and the hydrophobic interactions between the test ion and membrane substrate can reasonably be regarded as the “attractive” force [48].

Additionally, Equation (6) may also be used to determine the Gibbs free energy between the ion and the circle (ΔG_Ion-circle_). In this work, the nanopore (or circle) is simply regarded as a sphere with the radius R_Pore_. Of course, ΔG_Ion-circle_ is also dependent on the distance between them. This may be reasonably expressed as

(10) $$\Delta G_{Ion-circle}\propto 1/d_{Ion-circle}=1/\left(R_{Pore}+r_{Ion}+sep\right)$$

where d_Ion-circle_ is the distance between the centers of the ion and the circle (or nanopore), R_Pore_ is the pore radius, r_Ion_ is the ion size, and sep is the separation between the ion and the circle (Figure 2). Therefore, when the ion approaches the nanopore, the Gibbs energy of interfacial water may be related to the pore radius (R_Pore_), ion size (r_Ion_), and the separation between them. However, different from the ΔG_Ion-membrane_, the hydrophobic interactions between the ion and the circle (ΔG_Ion-circle_) may be the “attractive” or “repulsive” forces, which may be closely related to the ion size and pore radius.

As the ion and filter are embedded into water, hydration free energy is the sum of the Gibbs energy of water and interfacial water, and the latter is composed of the interactions between the ion and membrane substrate (ΔG_Ion-membrane_), and those between the ion and the circle with radius R (ΔG_Ion-circle_). From Equation (10), it can be seen that a decrease in the d_Ion-circle_ may increase the ΔG_Ion-circle_. In our recent works on hydrophobic interactions [47,48], dc (or R_c_) was expected to be

(11) $$\begin{array}{l} \Delta G_{Ion-membrane}-\Delta G_{Ion-circle\;\;(d=dc)}=\Delta G_{Water-water}\\ \left(Corresponding\;R_{Pore,c}\;and\;r_{Ion,c}\right) \end{array}$$

From the equation, dc is expected as the ion and channel come into contact (or with the separation between them being zero, sep = 0). Therefore, as an ion enters the channel, the dc may be closely related to the ion size (r_Ion_) and the radius of the channel (R_Pore_). Regarding an ion with a specific size r_Ion_, the corresponding pore radius (R_Pore,c_) may be expected for the ion, R_Pore,c_ for r_Ion,c_ = r_Ion_, or vice versa. Based on the discussion on hydrophobic interactions, with reference to dc, hydrophobic interactions between the ion and filter may be either “repulsive” or “attractive” forces when the ion approaches the filter.

**Figure 3:**
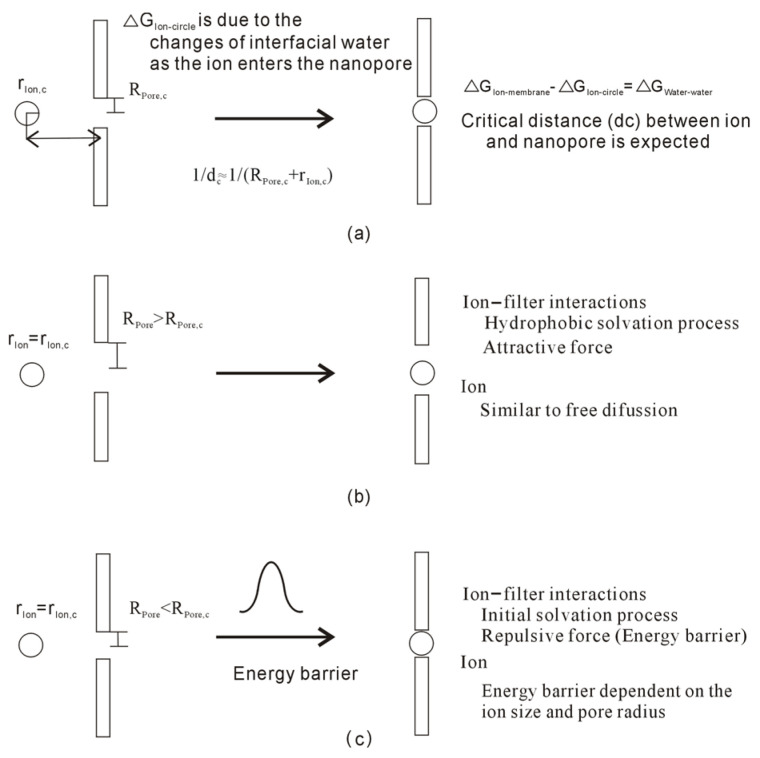
(**a**) Regarding the hydrophobic interactions between ion and filter, the critical distance (dc) can be expected, which is related to the pore radius (R_Pore,c_) and ion size (r_Ion,c_). (**b**) For the specific ion, the “attractive” force between the ion and filter is expected as the pore radius is larger than R_Pore,c_. (**c**) As the specific ion approaches the nanopore, the “repulsive” force is expected as the pore radius is less than R_Pore,c_. It seems that there is an energy barrier between the ion and nanopore.

Regarding the specific ion with radius r_Ion_, as the radius of the pore is larger than R_Pore,c_ (R_Pore_ > R_Pore,c_ and r_Ion_ = r_Ion,c_), it is associated with the hydrophobic process. Therefore, as the ion approaches the filter, an “attractive” force may be expected between them, and no energy barrier is expected (Figure 3). In other words, the ion is expected to penetrate the pore of the filter, similar to the ion diffusion in aqueous solutions.

Additionally, as the radius of the nanopore is less than R_Pore,c_ (R_Pore_ < R_Pore,c_ and r_Ion_ = r_Ion,c_), it is related to the initial solvation process. In the initial solvation process, the “repulsive” force between solutes is expected as they pushed together in water. In other words, as the ion approaches the nanopore, the energy barrier may be expected between the ion and the filter (Figure 3).

From the above, as the ion approaches the nanopore, the energy barrier between the ion and the channel may be related to the difference in Gibbs energy between ΔG_Ion-circle_ and ΔG_Ion-circle_ (d = dc). This may be expressed as

(12) $$\begin{array}{l} \Delta G_{Energy\;barrier}=\Delta G_{Ion-circle}-\Delta G_{Ion-circle\;\;(d=dc)}\\ \;\;\;\;\;\propto 1/\left(R_{Pore}+r_{Ion}\right)-1/\left(R_{Pore,c}+r_{Ion,c}\right) \end{array}$$

Therefore, when an ion approaches the nanopore, the energy barrier between them is related to the ion size (r_Ion_) and pore radius (R_Pore_).

**Figure 4:**
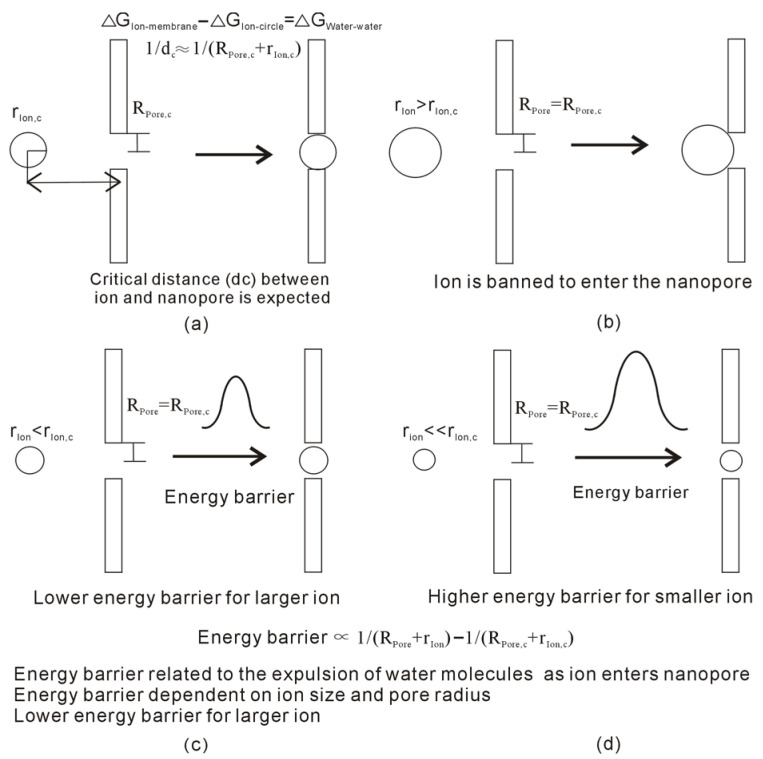
(**a**) As the ion approaches the nanopore, dc can be expected. (**b**) The ion is banned from passing through the nanopore when the ion is larger than r_Ion,c_. (**c**,**d**) When the ion enters the channel with a specific radius, the energy barrier is expected when the ion size is less than r_Ion,c_. For the specific nanopore with radius R, the energy barrier is related to the ion size. A lower energy barrier is expected for a larger ion.

Additionally, regarding the specific pore with the radius being R_Pore_, the energy barrier may also be expected as the ion size is less than r_Ion,c_, R_Pore_ = R_Pore,c_ and r_Ion_ < r_Ion,c_. For the filter with specific pore radius (R_Pore_ = R_Pore,c_), it can be derived that the energy barrier may be dependent on the ion size (r_Ion_). In comparison with r_Ion,c_, the lower energy barrier may be expected for the larger ion, and the higher energy barrier is related to the smaller ion (Figure 4). This may be utilized to understand the ion selectivity of the nanopore.

When the ions permeate the KcsA K^+^ channel, they become essentially dehydrated to pass through the narrow selectivity filter. Therefore, as the ion enters the nanopore, direct interactions, such as van der Waals interactions, are expected between the ion and nanopore. This means that the energy barrier may also be affected by the intermolecular interactions between the ion and nanopore. In other words, the ion selectivity of the nanopore may be modulated through changing the molecular polarity of the channel. In fact, this can be demonstrated with many theoretical simulations [31,32].

As ion size is less than r_Ion,c_ (r_Ion_ < r_Ion,c_ for R_Pore_ = R_Pore,c_), an energy barrier is expected between the ion and filter, and the ion may be suppressed to enter the nanopore of the filter. In other words, the external driving force, such as electrical potential or pressure, is necessarily added to the ion to surmount the energy barrier and pass through the nanopore. Of course, this is in accordance with the experimental studies on ion channel of the filter, which means that the electrical transmembrane potential is necessary inside and outside of the membrane to drive the ion through the channel.

From the above, the energy barrier between the ion and nanopore is related to ion size and pore radius. Regarding the nanopore with specific radius R_Pore_ (R_Pore_ = R_Pore,c_), a lower energy barrier is expected for large ions, and a higher energy barrier is related to small ions. Therefore, compared to small ions, the weaker external force may be enough to make large ions pass through the nanopore. This means that the ion selectivity of nanopores may also be related to the strength of external forces.

When the ion approaches the nanopore, “repulsive” or “attractive” forces may be expected between the ion and nanopore. From the above, dc is related to not only the ion size (r_Ion_) but also the radius of nanopore (R_Pore_). Regarding the nanopore with specific R_Pore_ (R_Pore_ = R_Pore,c_), the corresponding critical ion size (r_Ion,c_) may be expected. As ion size is less than r_Ion,c_, the energy barrier between the ion and nanopore is expected, r_Ion_ < r_Ion,c_ and R_Pore_ = R_Pore,c_. With reference to r_Ion,c_, the lower energy barrier may be expected for large ions. Additionally, as the ion passes through the filter, the direct interactions between the ion and channel, such as van der Waals interactions, become important, thereby influencing the energy barrier. In addition, the external driving force is necessarily added to the ion so that it can pass through the filter. This may be utilized to understand the ion selectivity of nanopores.

### 2.2. MD Simulations

From the above discussion, the ion selectivity of nanopores is closely related to the energy barrier as the ion enters the nanopore of the filter. Furthermore, the energy barrier between the ions and nanopore is related to both ion size and the radius of the pore. To understand the dependence of ion selectivity on the ion size, different ions (K^+^ and Na^+^) are forced to pass through the specific nanopore with a radius of 3.7 Å. In addition, to investigate the effects of nanopore pore radius on ion selectivity, MD simulations are also conducted on the systems in which a K^+^ ion enters the nanopores with different radii, such as 1.8 Å, 3.7 Å, and 5.6 Å.

**Figure 5:**
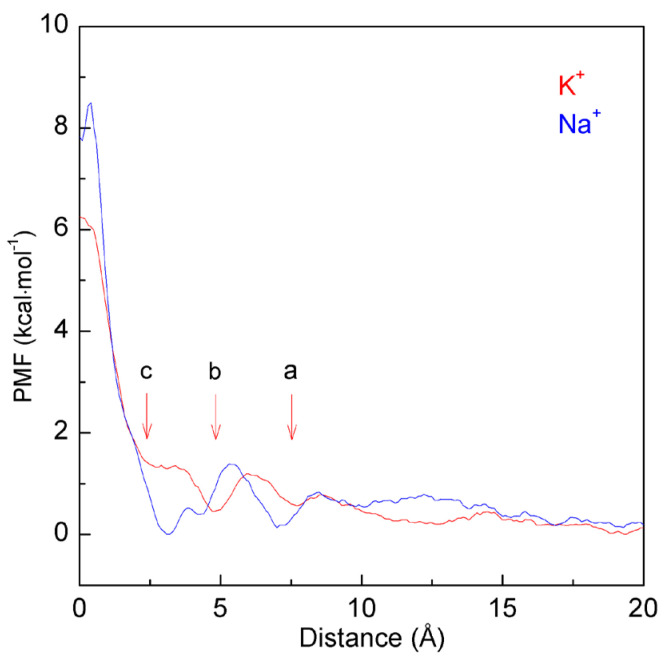
The PMFs as K^+^ and Na^+^ permeate the nanopore with a radius of 3.7 Å. During K^+^ approaches the nanopore, various minima are found at 7.5 Å, 4.7 Å, and 2.4 Å, which are labeled as a, b, and c. Compared with Na^+^, a lower energy barrier is found as K^+^ enters the nanopore. Due to the difference in energy barrier, this leads to the ion selectivity for K^+^ over Na^+^.

**Figure 6:**
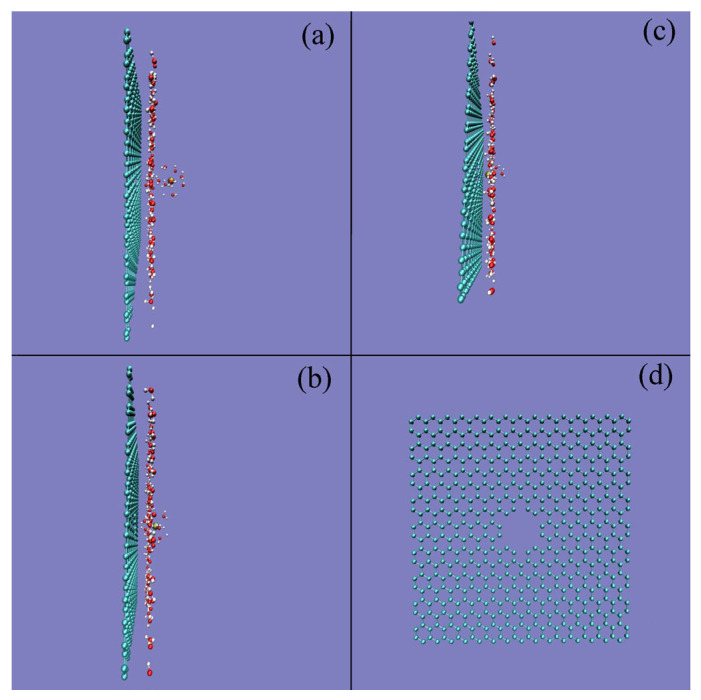
Snapshots of water molecules between K^+^ and the nanopore at various distances shown in Figure 5: (**a**) 7.5 Å, (**b**) 4.7 Å, and (**c**) 2.4 Å. (**d**) The nanopore with a pore radius of 3.7 Å is also shown.

In this work, the PMFs are calculated through ABF methods as a K^+^ ion perpendicularly approaching the nanopore with a radius of 3.7 Å (Figure 5). With a decrease in the distance between the K^+^ ions and nanopores, various minima can be found on the calculated PMFs (Figure 5). The minimum at 7.5 Å is found in the PMF, corresponding to a double water molecular layer located between the ion and nanopore (Figure 6a). Another minimum is located at 4.7 Å, attributed to the solvent-separated PMF, which means that only a water molecular layer can be found between the K^+^ ion and the graphene (Figure 6b).

From the calculated PMFs at 300 K, with decreasing distance between the K^+^ ion and graphene embedded with nanopores, energy barriers can be found between the neighboring minima (Figure 5). Of course, they may be related to the expulsion of a single water layer in the confined volume as the solutes moved closer together. Additionally, an obvious energy barrier can be found on the calculated PMFs as the distance between the K^+^ ions and nanopores is less than 5.0 Å, in which only one water layer can be found between them (Figure 6b), especially the distance less than 2.4 Å where K^+^ lies at the graphene surface (Figure 6c). Due to the obvious energy barrier between K^+^ ion and graphene, the ion is suppressed (or forbidden) from entering the nanopore of graphene (Figure 6d).

In addition, as the ion enters the nanopore, no water molecules can be found between the K^+^ ion and the nanopore of graphene, and the ion comes into contact with the graphene. This indicates that the energy barrier between the K^+^ ion and the nanopore may be closely related to the expulsion of interfacial water when the ion enters the nanopore. In other words, the above energy barrier may be related to the water molecules changing from interfacial to bulk water as the K^+^ ion approaches the nanopore of graphene. In combination with our recent studies [47,48], it may be concluded that this is closely related to hydrophobic interactions between the ion and nanopore.

Based on the above discussion on hydrophobic interactions, the dissolved K^+^ ion and graphene may be dispersed in water in the initial solvation process. To be more thermodynamically stable, water molecules can be expected between the K^+^ ion and graphene. In other words, it seems that there is a “repulsive” force as the K^+^ ion enters the filter, in which no water molecules can be found between them. Therefore, when the K^+^ ion enters the nanopore, the energy barrier may be due to the expulsion of interfacial water between the K^+^ ion and the graphene, and the K^+^ ion comes into contact with graphene in solutions. Of course, this is also in agreement with the above thermodynamic analysis on the energy barrier.

In our Raman spectroscopic studies [42,43,44], the Raman OH vibrations are mainly dependent on the local hydrogen-bonded network of a water molecule as three-dimensional hydrogen bondings appear in the water. Therefore, in ambient conditions, the dissolved solute mainly affects the structure of interfacial water. Of course, this is demonstrated by other studies on the structure and dynamics of water around ions using neutron and X-ray diffraction [53], X-ray absorption spectroscopy [54], femtosecond time-resolved infrared (fs-IR) vibrational spectroscopy [55,56,57], and optical Kerr effect spectroscopy [58]. These mean that the effects of ions on water may be largely limited to the first solvation shell.

**Figure 7:**
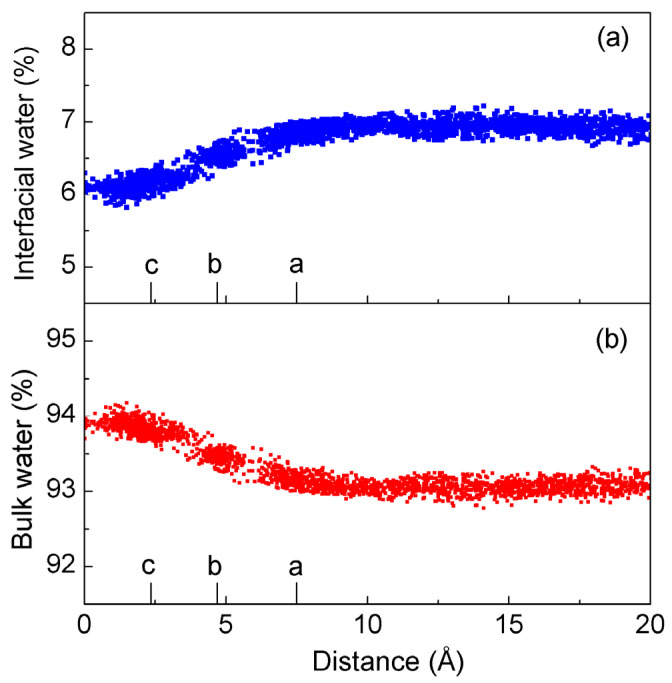
The changes in interfacial and bulk water as K^+^ passes through the nanopore. The letters a, b, and c mean the various distances between K^+^ and nanopore as shown in Figure 5.

The dissolved solutes primarily affect the structure of interfacial water. When they are dissolved in water, they can be divided into interfacial and bulk water. To investigate the structural reorganization between interfacial and bulk water, they are calculated as the K^+^ ion permeates the nanopore (Figure 7). With the decrease in the distance between the ion and nanopore, there is a decrease in the molecular number for interfacial water (Figure 7a), but an increase in the number for bulk water (Figure 7b). When the K^+^ ion enters the nanopore, the interfacial water associated with the ion and nanopore may be expelled into bulk water. Additionally, this is also related to the appearance of the energy barrier between the ion and nanopore.

To investigate the changes in hydrogen bondings when the K^+^ ion is forced to enter the nanopore of graphene, the hydrogen bondings of water are also determined. In this study, the geometrical definition of hydrogen bonding is utilized to determine the hydrogen bondings in water [59]. According to the geometrical definition, hydrogen bonding is considered to exist between two neighboring water molecules if the oxygen–oxygen distance (r_OO_) and ∠OOH angle between two water molecules are less than 3.5 Å and 30°, respectively.

**Figure 8:**
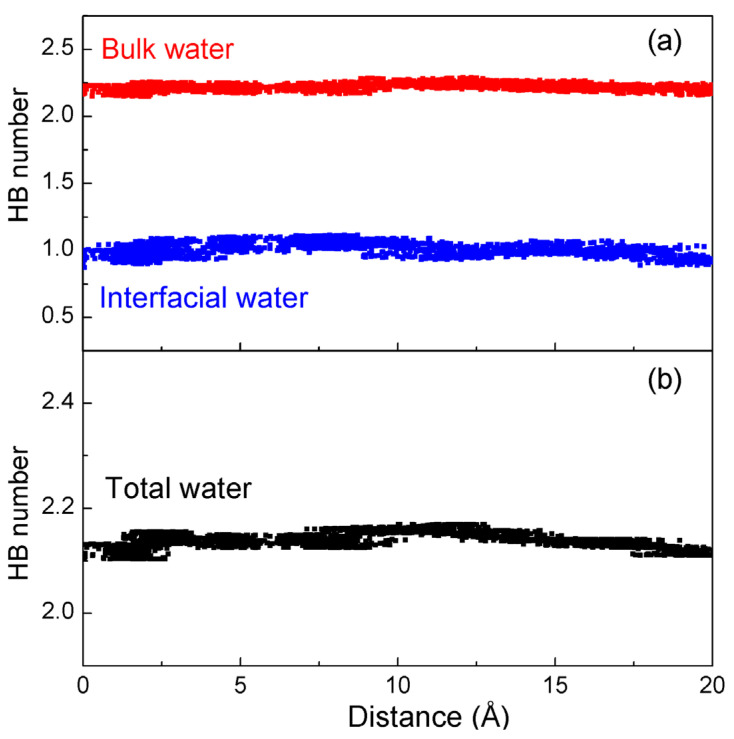
The hydrogen bondings number per water molecule in: (**a**) interfacial water and bulk water, and (**b**) total water as K^+^ permeates the nanopore.

From the MD simulations, the hydrogen bonding number (average number of hydrogen bonds per water molecule, n_HB_) can be calculated (Figure 8). In comparison with bulk water, due to the truncations of hydrogen bonding at the solute/water interface, the hydrogen bonding of interfacial water is less than that of bulk water (Figure 8). Therefore, the origin of hydrophobic interactions is due to the competition between the hydrogen bondings of bulk and interfacial water.

Regarding the nanopore with specific radius R_Pore_, the energy barrier between the ion and nanopore is expected as the ion size is less than r_Ion,c_, r_Ion_ < r_Ion,c_ and R_Pore_ = R_Pore,c_. Additionally, the above energy barrier may be dependent on the ion size. To understand the dependence of the energy barrier on the ion size, MD simulations are carried out on different ions (K^+^ and Na^+^) passing through the specific nanopore with a radius of 3.7 Å. From the calculated PMFs, the obvious barrier can also be found as the distance between the Na^+^ ion and the filter is less than 3.1 Å (Figure 5). However, compared to the Na^+^ ion, a lower energy barrier can be expected as the K^+^ ion enters the nanopore.

From the above discussion, the origin of the energy barrier between the ion and nanopore is closely related to the expulsion of the interfacial water layer between them as the ion enters the nanopore. This is in accordance with the fact that the ion will lose the hydration shell and become essentially dehydrated as the ion enters the pore. The dissolved ion mainly affects the structure of the interfacial water, and the coordination number within the first hydration shell is related to the ion size. Regarding Na^+^, the number of water molecules in the first hydration layer is 5.4, which is less than the corresponding number of K^+^ (8.0). This may be applied to understand the dependence of the energy barrier on ion size.

Due to the energy barrier, when the ion enters the nanopore, the ion may be suppressed or forbidden from entering the channel. Therefore, the external driving force, such as the transmembrane electrical voltage or pressure, is necessarily added to the ion so that it may surmount the energy barrier and pass through the filter. Regarding the channel with a specific radius pore R_Pore_, the energy barrier is dependent on the ion size. Under the same electrical voltage, it can be expected that the nanopore preferentially allows the specific ion with the lower barrier to pass through it. In comparison with small ions, a lower energy barrier is expected for larger ions. Therefore, compared to the smaller Na^+^ ions, the nanopore prefers the larger K^+^ ions to pass through the channel. This may be utilized to understand the ion selectivity of nanopores, such as KcsA for K^+^ over Na^+^.

**Figure 9:**
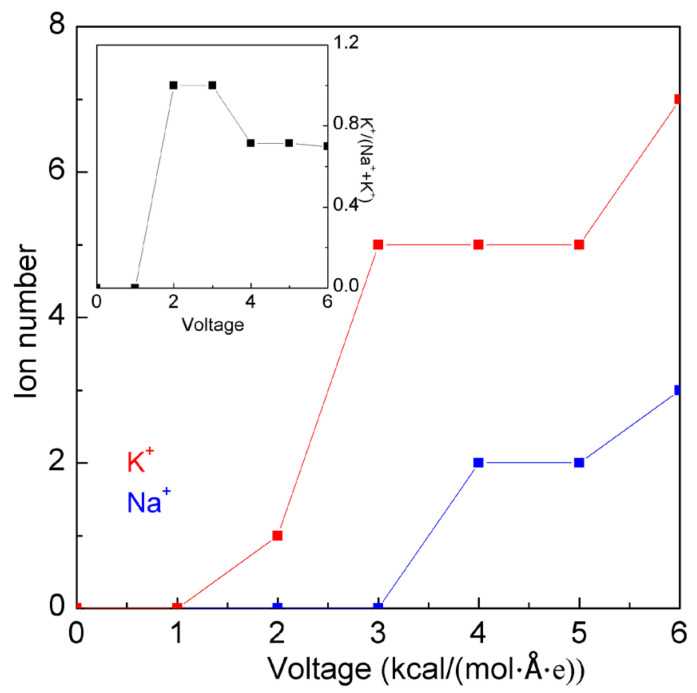
The effects of electrical voltage on the ion selectivity of a nanopore with a pore radius of 3.7 Å. The inlet shows the ion selectivity for K^+^/(K^+^ + Na^+^).

In this study, a few MD simulations were conducted to investigate the effects of electrical voltage on ion selectivity (Supplementary Materials). In the simulations, various electrical voltages were added to the aqueous solution, which was composed of 10 KCl and 10 NaCl molecules. It is necessary to provide enough voltage (2.0 kcal·mol^−1^·Å^−1^·e^−1^) so that the K^+^ ions may firstly pass through the filter with a pore radius of 3.7 Å (Figure 9). With increasing voltage, more K^+^ ions may pass through the filter. However, some Na^+^ ions were found to penetrate the nanopore at high voltages. As the voltage is 3.0 kcal·mol^−1^·Å^−1^·e^−1^, the filter gave the suitable ion selectivity of K^+^/(K^+^ + Na^+^) (Figure 9). Regarding the specific ion, it is important to design the corresponding nanopore for the ion. Additionally, it is also necessary to choose the reasonable voltage to drive the specific ion to pass through the corresponding nanopore of the filter.

**Figure 10:**
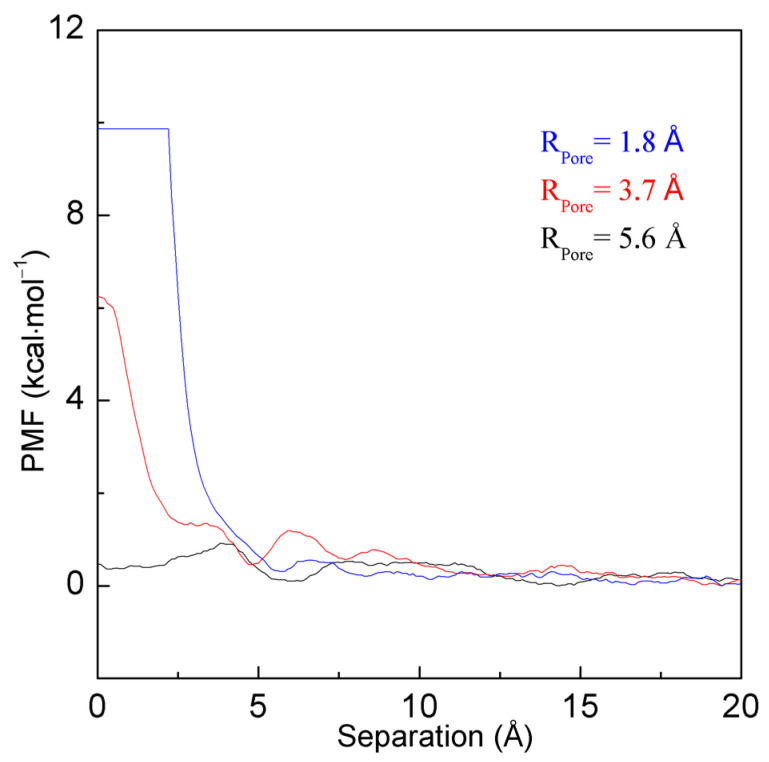
The PMFs as the K^+^ ion approaches the nanopores with different radii: 1.8 Å, 3.7 Å, and 5.6 Å.

In addition, the energy barrier between the ion and nanopore may be affected by not only the ion size but also the pore radius of the nanopore (R_Pore_ < R_Pore,c_ and r_Ion_ = r_Ion,c_). In this study, to investigate the dependence of ion selectivity on the radius of the nanopore, MD simulations were also conducted on the systems in which the K^+^ ion approaches the nanopore with different radii, namely 1.8 Å, 3.7 Å, and 5.6 Å. From the calculated PMFs (Figure 10), the energy barrier can be found as the K^+^ ion enters the nanopore with the different radii. However, with an increase in the radius of the nanopore, this decreases the energy barrier between the K^+^ ion and nanopore. This is in agreement with the above discussion on the effects of pore radius on the energy barrier.

From the calculated PMFs (Figure 10), a decrease in pore radius of the nanopore may lead to the increase in an energy barrier as the K^+^ ion enters the nanopore. Therefore, the strong transmembrane electrical voltage or driving pressure is necessarily added to the ion so that it can surmount the energy barrier and pass through the small nanopore. In fact, due to the strong energy barrier when K^+^ approaches the nanopore with a pore radius of 1.8 Å, the ion may be forbidden from entering the small nanopore even under high electrical voltage. Of course, the filter with this small pore radius is not suitably applied to separate the ion from others.

Additionally, an increase in the pore radius of the nanopore leads to a decrease in the energy barrier as K^+^ permeates the nanopore (Figure 10). In fact, no obvious energy barrier is found as K^+^ enters the nanopore with a pore radius of 5.6 Å. Therefore, the K^+^ ion may be expected to approach and freely enter the nanopore. In other words, this indicates that, with an increase in the pore radius, the hydrophobic interactions between the K^+^ ion and the nanopore may change from “repulsive” to “attractive” forces. Additionally, this also means that the nanopore with a pore radius of 5.6 Å may lose the ion selectivity for K^+^.

**Figure 11:**
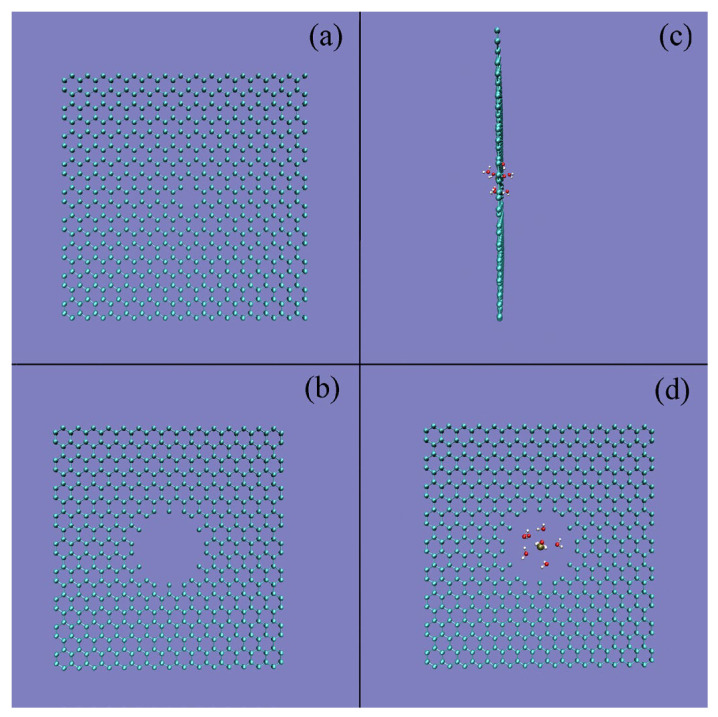
The nanopore with a pore radius of 1.8 Å (**a**) and 5.6 Å (**b**). (**c**,**d**) No obvious obstacle is found as K^+^ passes through the nanopore with a radius of 5.6 Å; this is because the interfacial water of K^+^ is preserved when it enters the nanopore.

From the above discussion, it can be stated that the ion selectivity of the nanopore is associated with the energy barrier between the ion and the nanopore, arising from the transformation of water molecules from interfacial to bulk water as the ion enters the nanopore. Therefore, if the interfacial water of the ion is still preserved when it passes through the channel, it may be expected that no energy barrier can be found. In fact, it can be found that the interfacial water of the K^+^ ion can still be found as K^+^ permeates the nanopore with a pore radius of 5.6 Å (Figure 11). In addition, this also indicates that the effects of the dissolved ions on water structure may be mainly limited to the first hydration shell of the ion.

**Figure 12:**
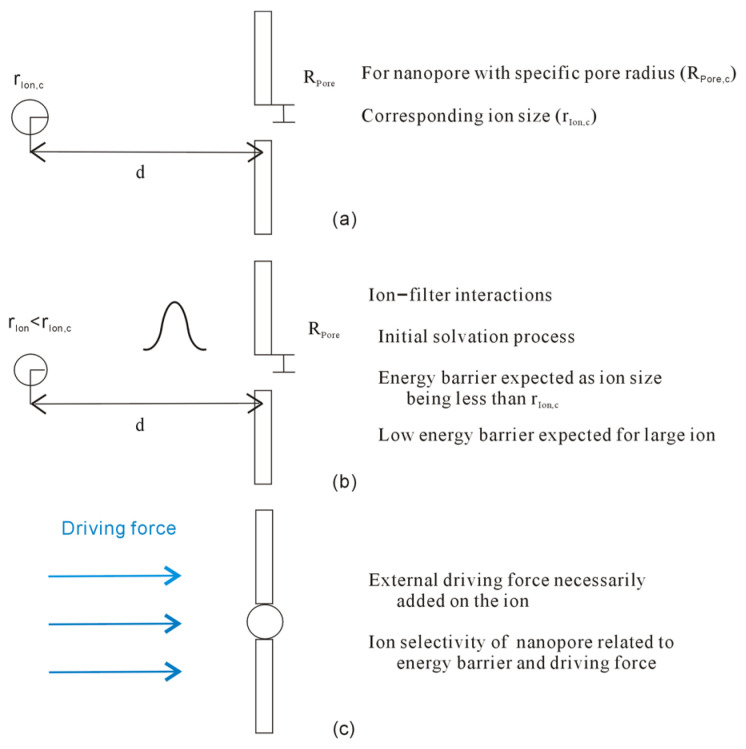
The mechanism of ion selectivity of nanopore. (**a**) In combination with our recent studies, the critical pore radius (R_Pore,c_) and ion size (r_Ion,c_) are expected during the ion approaches the nanopore. (**b**) During the ion enters the filter, energy barrier is expected as ion size being less than r_Ion,c_. (**c**) External force is necessarily added on the ion so that it enters the nanopore.

The ion selectivity of the nanopore is due to the difference in the energy barrier as various ions pass through the channel. Thermodynamically, the nanopore may prefer the specific ion with a lower barrier to permeate the filter. From this study, it can be derived that the energy barrier is closely related to the initial solvation process in which the Gibbs free energy of interfacial water is lower than that of bulk water. As the ions pass through the nanopore, they may become dehydrated. Therefore, the energy barrier is due to the water molecules transforming from interfacial to bulk water, which is dependent on not only the ion size but also the pore radius of the nanopore. Additionally, as the ion enters the channel, the energy barrier may also be affected by the direct interactions between the ion and nanopore. Due to the energy barrier between the ions and nanopore, the driving forces, such as the electrical voltage, may be necessarily added to the ions so that they may surmount the energy barrier and pass through the nanopore (Figure 12).

## 3. Method

### 3.1. Simulated Systems

The MD simulations were conducted using the program NAMD 2.12 [60]. The simulations were carried out on different systems, each containing a graphene filter embedded with a nanopore. The ion selectivity of the nanopore is related to the ion size and pore radius. After considering the pore radius of the KcsA channel, three nanopores with different radii were constructed: 1.8 Å, 3.7 Å, and 5.6 Å.

The empirical Chemistry at Harvard Macromolecular Mechanics (CHARMM) force field [61] was utilized to describe interatomic interactions. The water molecules were simulated using the intermolecular three-point potential (TIP3P) water model [62]. Non-bonded van der Waals interactions were smoothly switched to zero between 10 and 12 Å. The electrostatic interactions were simulated using the particle mesh Ewald (PME) algorithm.

In this study, the MD simulations were carried out in the NVT ensemble. The simulated temperature was 300 K, employing moderately damped Langevin dynamics. The simulated box was 40 Å × 40 Å × 60 Å. In addition, periodic boundary conditions were utilized in the three directions of Cartesian spaces. In the work, PMFs were determined through the adaptive biasing force (ABF) calculations [63,64,65,66,67,68]. The total time for ABF simulations was 80 ns. Additionally, a few simulations were also performed to study the ion selectivity of nanopores under various electrical voltages (Supplementary Materials). For MD simulations under electrical voltages, the total simulated time was 84 ns. Additionally, the simulated results were analyzed through VMD package [69].

### 3.2. ABF Calculations

In this work, PMFs were calculated to investigate the mechanism of ion selectivity of nanopores. These were carried out using NAMD with ABF [63,64,65,66,67,68] extensions integrated in the Collective Variables (CVs) module [70].

The ABF method is based on the thermodynamic integration of average force acting on coordinates, which is a combination of probability density and constraint force methods. In the ABF method, a biasing force opposing the actual force arising from system components is periodically applied to the reaction coordinate to generate what is effectively a random walk along the reaction coordinate (purely diffusive dynamics). For the ABF method, the free energy along a transition coordinate can be seen as a potential arising from the average force acting along the coordinate.

In this study, the target nanopore is fixed, and the test cation (K^+^ or Na^+^) is restrained to perpendicularly move to the center of graphene nanopore along Z axis. In the ABF calculations, the distances between the ions and nanopores are regarded as CVs, which are broken down into several consecutive windows, each with 2.5–2.7 Å wide.

## 4. Conclusions

Based on thermodynamics analysis and MD simulations, this work is devoted to investigating the mechanism of ion selectivity in nanopores. From the work, the following conclusions are derived:

(1) The ion selectivity of the channel is related to the rearrangement of water molecules as the ion enters the channel. In combination with our recent studies, the ion selectivity of the nanopore may be related to hydrophobic interactions between the ion and nanopore.

(2) In our recent works on hydrophobic interactions, with reference to Rc, it was divided into initial and hydrophobic solvation processes. In the hydrophobic process (>Rc), the ions are expected to freely pass through the nanopore (r_Ion_ = r_Ion,c_, R_Pore_ > R_Pore,c_) or are forbidden from passing through the channel (r_Ion >_ r_Ion,c_, R_Pore_ = R_Pore,c_). It seems that there are the “attractive” forces between the ions and nanopore.

(3) In the initial process (<Rc), the energy barrier can be expected as the ion enters the channel (r_Ion<_r_Ion,c_, R_Pore_ = R_Pore,c_), which is related to the ion selectivity of the nanopore. It is found that the energy barrier is related to the ion size and pore radius. Additionally, the ion selectivity of the nanopore is also affected by the external forces added to the ions to overcome the energy barrier.

## Data Availability

Data are contained within the article and Supplementary Materials.

## Supplementary Materials

The following supporting information can be downloaded at: https://www.mdpi.com/article/10.3390/molecules29040853/s1, A few MD simulations were performed to study the ion selectivity of the nanopore under various electrical voltages. Some simulated trajectories are provided in the Supplementary Data.
