# Supplementary material for: The Molecular Mechanism of Ion Selectivity in Nanopores
Source: Molecules. 2024 Feb 14;29(4):853. doi: 10.3390/molecules29040853 (PMC10891634; doi:10.3390/molecules29040853)
Supplement: Supplementary file 1 [file molecules-29-00853-s001.zip › molecules-2736645-supplementary-R.pdf]

Supplementary Materials

The Molecular Mechanism of Ion Selectivity in Nanopores

Yan-Nan Chen, Yu-Zhen Liu and Qiang Sun*

Key Laboratory of Orogenic Belts and Crustal Evolution, Ministry of Education,

The School of Earth and Space Sciences, Peking University, Beijing 100871, China; 2301210129@stu.pku.edu.cn (Y.C.); liuyuzhen@stu.pku.edu.cn (Y.L.)

* Correspondence: QiangSun@pku.edu.cn

In this study, a few MD simulations were performed to study the ion selectivity of nanopore under various electrical voltages. MD simulations were conducted using NAMD 2.12 package. The simulations were carried out in the NVT ensemble. The simulated temperature was kept at 300 K. During the simulations, the empirical CHARMM force field was utilized to describe interatomic interactions. The water molecules were simulated using the intermolecular three point potential (TIP3P) water model. For each MD simulation, the simulated time was 12 ns, and the time step was 2 fs. The simulated results are analyzed through VMD package.

In the simulations, a graphene sheet and a filter are embedded into a water box (40 Å×40 Å×60 Å). Regarding the filter, it is a graphene sheet embedded with a nanopore with radius being 3.7 Å. During the simulations, 10 KCl and 10 NaCl are embedded between the filter and sheet. Due to the energy barrier between the ions and nanopore, the ions are forbidden to pass through the filter (SI-1, voltage is 0). To make the ions to penetrate the filter, electrical voltage may be added on the systems. It is found that enough voltage (2.0 $kcal \cdot mol^{-1} \cdot Å^{-1} \cdot e^{-1}$) is necessarily added on the system so that K^+ ions may pass through the filter. With increasing voltage, more K^+ ions may pass through the filter (SI-2, voltage is 3). However, some Na^+ ions are found to penetrate the nanopore at high voltage (SI-3, voltage is 6). As the voltage is 3.0 $kcal \cdot mol^{-1} \cdot Å^{-1} \cdot e^{-1}$, the filter gives the suitable ion selectivity of $K^+/(K^++Na^+)$ (SI-2, voltage is 3). Regarding the specific ion, it is important to design the nanopore with suitable pore radius. Additionally, it is also necessary to choose the reasonable voltage to drive the specific ion to pass through the corresponding nanopore. Some simulated trajectories are provided in supplementary data, such as SI-1, SI-2, and SI-3. In the trajectories, only K^+ and Na^+ ions are shown.
